# The Value of Exercise1Read at the Keystone Veterinary Medical Association, October, 1897.

**Published:** 1897-12

**Authors:** J. Curtis Michener

**Affiliations:** Colmar, Pa.


### THE VALUE OF EXERCISE.[1]

By J. Curtis Michener, V.S.,
COLMAR, PA.

I come before you this evening more for the purpose of apology than to fulfil the appointment with which you have honored me. By a misunderstanding I expected this meeting to occur upon the last Tuesday of the month (26th), and had not commenced the preparation of a paper when receiving notice last Saturday.

[1] Read at the Keystone Veterinary Medical Association, October, 1897.

The time being so short, and having so much else on hand, I have merely scribbled off a few thoughts. I beg you to excuse the effort and the seeming impertinence of addressing a body of medical men upon the value of exercise, when it is already conceded by you all. It is not lack of knowledge so much as failure to practice, that has convinced me that our profession, and medical brothers as well, need severe criticism. Exercise out in the open air and sunlight is the most essential requisite, even necessity, to that robust health we all admire, and which gives the keenest appetite and enjoyment. By exercise the circulation and respiration are accelerated and deepened. The skin is made to glow and perspire, and tone and strength are given every organ. Daily exercise is necessary to promote the secretions, excretions, and exhalations. When neglected, stagnation and impurity begin, and, if habitual, disease is invited and life shortened. It is a fortunate provision of nature that most of us are compelled to be active, and she imposes a just punishment upon those who subsist upon the labor of others. Examples of the truthfulness of these statements are to be seen upon every hand. Indeed, they are so common and universal that we look upon them without seeing, and experience without realizing.

Sex makes but slight natural difference in health and strength, but our countrymen are far superior to the women in strength and health (in spite of rum and tobacco), simply because the women do not get the invigorating sunlight and pure air exercise. Many of your city chaps are quite effeminate from the same causes. I think I have done enough preaching, and will get down to veterinary practice.

Exercise has a hygienic and therapeutic value, both of which need to be better understood and utilized. It is impossible for any animal to long remain in a hardy, healthy condition, at the height of capacity and transmit a vigorous constitution, without regular and abundant exercise. Many a poultry man has come to grief by building warm quarters and keeping his stock too closely confined, instead of allowing them to forage around and scratch for a good part of their living.

Many think the only proper place for a hog is in a pen, which does seem to answer for the brief period that the average porker is allowed to live. But the breeder of swine who practises that system is sure to meet failure.

Cows stand confinement the best of any farm animals. In some instances they never leave the stall during the winter months, and appear to do very well, but their stock of vitality is necessarily much reduced, and ofttimes the way is paved for trouble and loss in the future. I was gratified to learn that Miller & Sibley's fine herd of Jerseys, which we so recently visited, have to walk one and a quarter miles up into the mountain to obtain their water every day of the year, and are driven around for exercise besides when not at pasture. That is rational treatment, and results in good health and staying qualities. Our horses suffer much from want of and irregularity of exercise. Strange it is that so many intelligent men suffer their horses to stand tied in the dark stable for several days in succession, and then require a full day's work, and wonder why they get sick and go blind.

It is of the therapeutic value of exercise that I feel moved to speak in particular, because our literature and practice indicate that its value is not recognized. Many abnormal conditions are the direct result of the lack of judicious exercise, and are most successfully removed by attention thereto. Swollen sheath and legs are better, more quickly and permanently cured by exercise than a resort to physic. Lymphangitis (after paying attention to the slight abrasion about the foot or leg, which can usually be found) should be cured by exercise. Greasy heel is very amenable to treatment when the patient is regularly worked.

The horse with strangles should never quit work, and he will make a far quicker and better recovery than if closely housed and drugged. The same applies to influenza. During our great epizooty the horses that were kept going did by far the best, while those closely stabled suffered the complications and relapses. When you have a case of dumb staggers see to it that he be made to work in the cool part of every day. In the winter freeze and work it out of him. In the early or congestive stage of laminitis blanket warmly and give prolonged exercise in a grass field while wet with dew. Chronic laminitis that has not resulted in much disorganization should be treated the same way. Pull his shoes off and put him to plowing in the furrow. Drive him on the muddy roads and in the snow. Atrophy of muscle is cured by putting to use, of course. Congestive pneumonia and chill are readily cured by warm clothing and exercise. Impaction of the bowels will usually yield to a three or five mile gallop. I do not expect many of you to adopt this treatment in these days of eserine and barium chloride, or to keep a case of flatulent colic moving, and lose the glory of performing paracentesis abdominalis. But do, please, exercise an ordinary case of indigestion in horse, or man, or cow.

I now wish to urge my panacea in cases of azoturia, knowing beforehand that many of you think it strongly contraindicated. We know that exercise provokes the attack; we also know want of exercise precedes and predisposes to the attack. I know that enforced continuous exercise will cure an ordinary case completely in a few hours. I never lost a case that was reached before going down. Bear in mind that this is a congestive disease. The blood has become thick and surcharged with impurities *from want of exercise.* The subject is taken from the stable feeling good. The atmosphere is damp and chilly. The skin is contracted. The capillary circulation is restricted. Exercise puts the heart to work rapidly and powerfully. The circuit becomes impeded. Heavy congestions of muscles, usually of the loins and shoulder, often becoming as hard as a board, take place, causing pressure upon the nerves, pain and approaching paralysis. If the patient be stopped in the early stages, sheltered and warmly clothed, this congestive process may be arrested and gradually overcome. I cannot speak from experience, as our practice has always been to rub the affected parts with strong turpentine liniment, clothe warmly, and keep in motion until the danger-point is passed, which is usually within two hours. I will relate a case as an illustration. While a young man at home, my father owned a fast trotting young mare. He started on a trip to Boston, to be gone ten days. A deep snow had fallen, and as I took him to the depot, his last stern command was: "Don't take Polly out while I am gone. She will jump into some of these drifts and upset, and I would not have her run away for five hundred dollars." Sunday came, and wishing to give my best girl a sleigh-ride with the best horse, I could not resist the temptation. The mare felt good, and just flew along like a bird for the first mile and a half, when she commenced to slacken, step short, and quiver in the flanks and shoulders. Awful visions of paternal wrath flashed across the horizon of my frightened imagination. No time to lose, I sprang from the sleigh, stripped off the harness, put on blankets and robe, and tried to lead her; no go. Took the lines behind her, and with whip in hand cut her around the legs without mercy. Every few steps it would seem she must go down and all hopes go down with her. Renewed chastisement would send her on again. When about half-way home I was more than gratified to notice some improvement; by the time home was reached she walked much better. I continued to move her along the warm side of barn one hour longer, when she was entirely relieved. Have worked that plan in many cases with complete and speedy success. A case of azoturia should never stop moving until well. If stopped, they go down, and if once down usually die. Judicious exercise is very beneficial to the convalescing cases of about all diseases except diarrhœa and pleurisy.

Sunlight is a great source of invigoration. We should give our patients sun-baths protected from the winds. We should always bear in mind that no animal can have perfect health, or be restored when sick, if confined in a dark place without exercise.

